# Fabrication and Characterization of the Egg-White Protein Chitosan Double-Layer Emulsion

**DOI:** 10.3390/molecules27186036

**Published:** 2022-09-16

**Authors:** Qingmei Pan, Zhipeng Zhang, Ruifang Wu, Danjun Guo, Wei Xu, Hongxun Wang, Yang Yi

**Affiliations:** 1 School of Food Science and Engineering, Wuhan Polytechnic University, Wuhan 430023, China; 2 Key Laboratory for Deep Processing of Major Grain and Oil, Ministry of Education (Wuhan Polytechnic University), Wuhan 430023, China; 3 BG (TianJin) Grain and Oil Industry Co., Ltd., Tianjin 300452, China

**Keywords:** egg-white protein, chitosan, particles, double-layer emulsion, characterization

## Abstract

Egg-white protein has an abundance of hydrophobic amino acids and could be a potential emulsifier after modification. Here, egg-white protein was modified via ultrasonic and transglutaminase treatments to destroy the globular structure. The egg-white protein gel particles (EWP-GPs) were prepared and then a novel highly stable EWP-chitosan double-layer emulsion was constructed. When ultrasonic treatment was applied at 240 W and TGase (20 U/g EWP) treatment, the EWP-GPs had a low particle size and good emulsification performance. The particle size of EWP-GPs was a minimum of 287 nm, and the polymer dispersity index (PDI) was 0.41. The three-phase contact angle (θ_o/w_) of EWP-GPs was 79.6° (lower than 90°), performing with good wettability. Based on these results, the EWP-chitosan double-layer emulsion was prepared through the EWP-GPs being treated with 240 W ultrasound, TGase, and chitosan in this study. When the double-layer emulsion had 0.6% (*v/v*) chitosan, the zeta potential of the double-layer emulsion was −1.1 mV and the double-layer emulsion had a small particle size (56.87 µm). The creaming index of double-layer emulsion at 0.6% (*v/v*) chitosan was 16.3% and the droplets were dispersed uniformly. According to the rheological results, the storage modulus (G′) was larger than the loss modulus (G″) in the whole frequency, indicating the formation of an elastic gel network structure in the emulsion. It is hoped to develop a novel food-grade stabilizer and a stable double-layer emulsion, providing new environment-friendly processing in hen egg products and delivery systems.

## 1. Introduction

Emulsion is a common material for delivering bioactive substances in food, pharmacy, and cosmetics [1], while single-layer emulsion is usually limited by its instability to environmental change [2]. Recently, double-layer emulsion has become a hot issue for avoiding instability constraints on environmental conditions [3,4]. The double-layer emulsion was a water-in-oil-in-water (W_1_/O/W_2_) system and it was generally prepared in two steps [5]. The first step was to prepare a W_1_/O emulsion as the internal phase. Then, the W_1_/O emulsion was dispersed in the external aqueous phase (W_2_). It was reported that a W_1_/O/W_2_ emulsion should be stabilized with two kinds of emulsifiers (lipophilic to W_1_/O and hydrophilic to O/W_2_) to avoid the migration of the internal and external phases [6]. Additionally, a Pickering emulsion was stabilized through solid particles and exhibited high stability against coalescence [7]. Thus, Pickering emulsion is a potential internal phase to improve the stability of the double-layer emulsion [7,8,9]. Food-grade particles derived from proteins, polysaccharides, and lipids were reported to be used to stabilize the Pickering emulsions [10]. Among these particles, protein particles, as an excellent natural emulsifier, have high surface activity and good digestibility [11].

In recent years, many protein particles have been used to stabilize Pickering emulsions, such as whey protein isolate [12] and zein [13]. In fact, egg-white protein has superior gelation and is amphiphilic due to its abundant hydrophobic amino acids [14,15]. However, egg-white is a typical globular protein, and a large number of hydrophobic groups are embedded in the protein molecules and exhibit strong hydrophilic properties [16]. Protein modifications (chemical modification, physical modification, and enzymatic modification) could induce partial unfoldment and aggregation of globular proteins, so the hydrophobic groups could be exposed to the surface and show good emulsification. Among these modification methods, the ultrasonic and enzymatic modifications are safe, low-loss, and friendly to the environment [17]. In addition, ultrasonic modification combined with enzymolysis could improve the efficiency of protein modification and boost the biological activity of proteins [18]. Transglutaminase (TGase) is an acyltransferase, and it could boost the cross-linking and gelation properties of protein [19]. Studies found that ultrasound combined with TGase treatment was a superior processing technology which improved the solubility and emulsifying properties of protein particles, such as soy protein [20] and soybean-whey mixed protein [21]. However, protein particles may induce the aggregation of droplets and cause the instability of emulsions [22].

Chitosan, a natural polysaccharide, is the by-product of chitin deacetylation [23]. It has strong hydrophilic properties and good adhesion. Chitosan could change the viscosity of emulsions and provide repulsive electrostatics to prevent droplet aggregation [24]. A recent study found that whey protein isolate and chitosan hydrochloride were used to prepare double-layer emulsion via layer-by-layer self-assembly, which showed better stability than monolayer emulsions [25]. The purpose of this study was to obtain the EWP-GPs with good emulsification and construct a highly stable EWP-chitosan double-layer emulsion system. In this study, egg-white protein was first modified via ultrasonic and transglutaminase treatments, and EWP-GPs were prepared and characterized by particle size, fluorescent spectrometry, three-phase contact angle, and emulsification. Secondly, the EWP-chitosan double-layer emulsion was constructed and investigated through the particle size, zeta potential, rheology, and microstructures. This study is expected to expand the application of egg-white as a stabilizer and obtain a food-grade double-layer emulsion to deliver bioactive substances.

## 2. Results and Discussion

### 2.1. Preparation and Properties of Egg-White Protein Gel Particles

#### 2.1.1. Particle Size and Wettability

**Figure 1:**
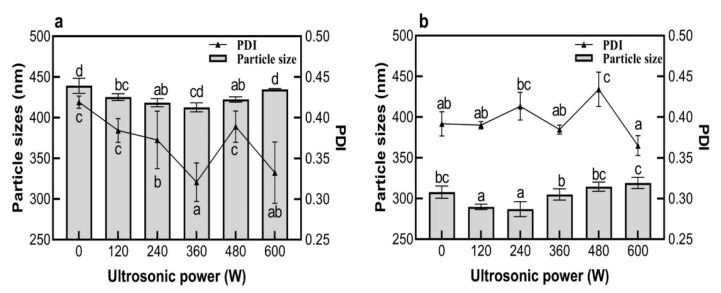
Effect of ultrasonic power on the particle size of EWP-GPs (**a**) and EWP-GPs with TGase treatment (**b**) of EWP-GPs. (Values with different letters are significant at *p* < 0.05).

The polymer dispersity index (PDI) was used to describe the molecular weight distribution of polymers [26]. As shown in Figure 1a, the particle size of EWP-GPs without ultrasonic treatment was 439.17 nm. When the ultrasonic power was 360 W, the particle size of EWP-GPs significantly decreased to the smallest size at 412 nm (*p* < 0.05), and the PDI was at a minimum of 0.32. The PDI of EWP-GPs with 360 W ultrasonic treatment was significantly lower than the EWP-GPs without ultrasonic treatment (*p* < 0.05), indicating that 360 W ultrasonic treatment could improve the dispersion and stability of EWP-GPs. This phenomenon might be attributable to the mechanical shear produced by the ultrasonic treatment [27]. A study prepared a flaxseed oil-in-water emulsion and found a similar phenomenon: with the improvement of the ultrasonic power (generation at 20–24 kHz), the mechanical shear was strengthened, and the particle size and PDI were decreased [28]. Overall, a 360 W ultrasonic treatment could provide EWP-GPs with good dispersibility.

Figure 1b showed the particle size of the EWP-GPs prepared under TGase treatment combined with differing ultrasonic power (0–600 W). The size of EWP-GPs prepared with TGase but without ultrasonic treatment was 307.83 nm, which was much smaller than the EWP-GPs without treatment (439.17 nm). This was because transglutaminase improved the jelly strength of egg-white protein and produced smaller particles in preparation [29]. With 240 W ultrasonic and TGase treatment, the EWP-GPs reached the smallest particle size (287 nm), and the PDI was 0.41. This phenomenon suggested that ultrasonic and TGase treatment could decrease the particle size of EWP-GPs and improve the stability of particles. As Figure 1a,b show, the PDI value of EWP-GPs with ultrasonic-TGase treatment was higher than that of EWP-GPs with ultrasonic treatment. This might be due to the jelly strength of EWP-GPs being improved by TGase treatment, and uneven particles were generated in preparation.

**Figure 2:**
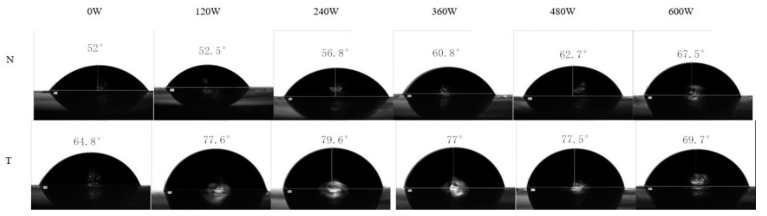
The effect of different treatments on the three-phase contact angle. (The EWP-GPs were treated with ultrasound from 0 W to 600 W, respectively. The “N” represents EWP-GPs without TGase treatment, and the “T” represents EWP-GPs with TGase treatment).

As a Pickering particle, the particle should be insoluble but partially wetted by the continuous and dispersed phases. Partial wettability of particles contributed to sufficient interfacial adsorption efficiency. A three-phase contact angle of particles was the angle formed at the three-phase boundary where the solid particles, the continuous phase, and the dispersed phase intersect. The wettability of EWP-GPs could be evaluated by the three-phase contact angle (θ_o/w_), which could evaluate the adsorption efficiency of the emulsion. When the θ_o/w_ was less than 90°, the particles showed a good hydrophilicity. When the θ_o/w_ was more than 90°, the particles were inclined to show a good hydrophobicity. When the θ_o/w_ was 90°, the particles had the strongest absorption and could prevent the aggregation of droplets [2]. As shown in Figure 2, the θ_o/w_ of EWP-GPs without any treatment was 52°: this indicated that the hydrophobicity of the particle was weak. The θ_o/w_ of EWP-GPs with 600 W ultrasonic treatment reached a peak (67.5°). These results indicated that the ultrasonic treatment could improve the hydrophobicity of EWP-GPs and that the EWP-GPs were partially wetted. It could be inferred that ultrasonic treatment caused the exposure of the protein chains and hydrophobic groups. A study suggested that ultrasonic power could destroy the non-covalent interaction of the whey proteins’ particles and enhance the hydrophobicity of particles [30]. The θ_o/w_ of EWP-GPs with 240 W ultrasonic and TGase treatment was the largest (79.6°). This result showed that ultrasonic and TGase treatment could improve the hydrophile and lipophile of EWP-GPs. Transglutaminase could catalyze the formation of covalent bonds between lysine and glutamic acid in protein amino acids [31]. In addition, the ultrasonic treatment could induce the protein to unfold and the hydrophobic groups’ exposure, resulting in facilitating the cross-link between an enzyme and a protein [32]. A previous study also found that ultrasound pretreatment promoted the enzymatic hydrolysis of soy protein isolates and then improved their surface hydrophobicity [31]. Overall, when EWP-GPs were treated with 240 W ultrasonic power combined with TGase, the particles performed a superior amphipathicity to prepare O/W emulsion.

#### 2.1.2. Zeta Potential of EWP-GPs and Chitosan

**Figure 3:**
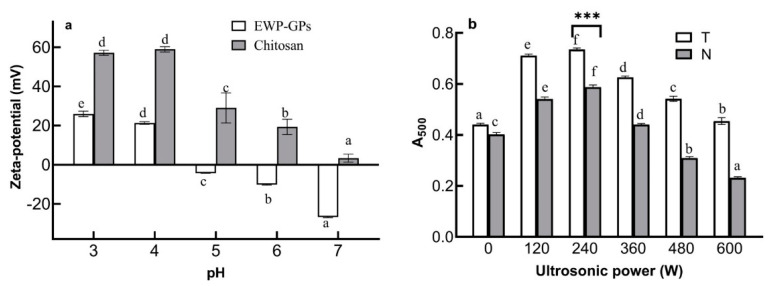
The effect of pH on the potential of EWP-GPs and chitosan (**a**). The emulsification of EWP-GPs (**b**). (Values with different letters are significant at *p* < 0.05, and *** represents *p* < 0.001).

The double-layer emulsion had higher stability and protein–polysaccharides (such as a chitosan compound which was a hot issue to prepare double-layer emulsion in recent years). Egg-white protein contains a variety of proteins, and the isoelectric point is between 4 and 5 [14]. Therefore, when the pH value was less than 5, the dispersion surface of EWP-GPs was positively charged. Chitosan is a cationic polysaccharide with positive charges in a wide range of pH values. In addition, the aqueous solution of chitosan is positively charged at pH 6.0 [33]. As Figure 3a shows, when the pH was 6.0, the zeta potential of EWP-GPs and chitosan solution were −10.18 mV and 19.33 mV, respectively. The zeta potential of EWP-GPs and the chitosan solution were opposite, and the values were both higher than 10 mV. It was suitable to prepare stable emulsions. A previous study found that opposite charges between zein and gum Arabic caused gum Arabic to absorb on the surface of zein particles [13]. Through mechanical stirring, the cationic polyelectrolyte chitosan was adsorbed to the emulsion interface by electrostatic interaction to form an ultra-thin polymer interfacial film [23]. In addition, the electrostatic attraction between EWP-GPs and chitosan could be used to prepare the double-layer emulsion. Therefore, we could adjust the pH value to 6 to prepare the double-layer emulsion.

#### 2.1.3. Emulsification of EWP-GPs

Emulsification indicates the ability of a protein to adsorb at the oil–water interface. As Figure 3b showed, when the EWP-GPs were treated with 240 W ultrasonic and TGase, the absorbance of EWP-GPs reached the maximum (0.74). In addition, the TGase significantly increased the emulsification of EWP-GPs with 240 W ultrasonic-TGase treatment (*p* < 0.001) compared with the EWP-GPs with 240 W ultrasonic treatment. It revealed that ultrasonic and TGase treatment could improve the emulsification of EWP-GPs. Ultrasonic and enzymatic treatment could increase the reaction sites and improve the emulsification of particles [34,35]. The emulsification of EWP-GPs was bound up with the surface hydrophobicity of EWP-GPs. A study found a similar phenomenon in Cyperus esculentus seed protein [36]. The EWP-GPs with 240 W ultrasonic-TGase treatment had high surface hydrophobicity and emulsification, and the particles could be used to prepare emulsions.

#### 2.1.4. Intermolecular Force

**Figure 4:**
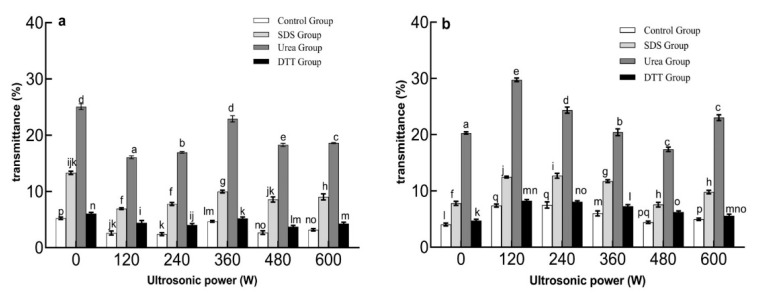
The transmittance of ultrasonic treatment EWP-GPs (**a**) and ultrasonic-TGase treatment EWP-GPs (**b**). (Values with different letters are significant at *p* < 0.05).

As Figure 4a shows, the transmittance of the particles in the control group presented irregular changes with the increase in ultrasonic power. These results were not consistent with the changes of particle size. The reason might be that the ultrasonic power promoted the aggregation of EWP-GPs. As Figure 4b shows, the transmittance of the particles in the control group with 240 W ultrasonic-TGase treatment reached the highest value (7.5%): this was consistent with the result of particle size. This also suggested that the smaller particles had higher transmittance. As Figure 4a,b show, the transmittances of EWP-GP dispersions were significantly increased with the addition of SDS, Urea, or DTT (*p* < 0.05). It was found that SDS, Urea, and DTT could destroy the hydrophobic interaction, hydrogen bond, and disulfide bond inside the particles, respectively [37]. Therefore, the hydrophobic interaction, hydrogen bond, and disulfide bond were the intramolecular interactive forces maintaining the internal structure of the EWP-GPs. When the EWP-GPs were untreated with ultrasound and TGase, the transmittances in Urea, SDS, and DTT groups increased by 10%, 5%, and 1% of particles compared with the transmittances of the control group, respectively. The results showed that the hydrogen bond was the main force to maintain the stability of EWP-GPs.

In conclusion, when the EWP-GPs were treated with 240 W ultrasound and TGase, the particles had small particles and good emulsification, which was suitable to prepare highly stable double-layer emulsions. In addition, when the pH value was 6, the EWP-GPs and chitosan were suitable to prepare the double-layer emulsion.

### 2.2. Characteristics of Double-Layer Emulsions

#### 2.2.1. Zeta Potential and Particle Size of the Double-Layer Emulsion

**Figure 5:**
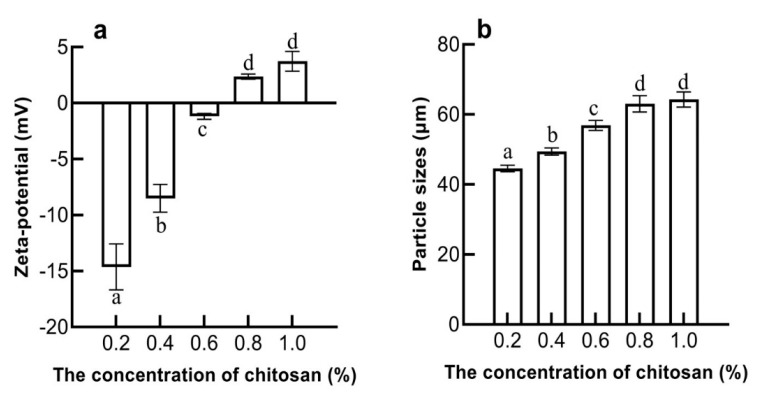
The effect of chitosan concentration on the potential of the double-layer emulsion (**a**). The effect of chitosan concentration on the particle size of the double-layer emulsion (**b**). (Values with different letters are significant at *p* < 0.05).

Figure 5a showed that when the concentration of chitosan was 0.6%, the positive and negative charges on the surface of the emulsion tended to balance, and the zeta potential of the double-layer emulsion was −1.1 mV. When the pH was 6, the potential of EWP-GPs was −10.18 mV and the potential of chitosan solution was 19.33 mV. With the increase in the concentration of chitosan, the zeta potential of the double-layer emulsion changed from negative to positive. Therefore, it could be inferred that the chitosan absorbed on the surface of EWP-GPs in the emulsion. A study found similar results: that the chitosan molecules could be adsorbed on the surface of WPI gel particles and the zeta potential changed with the increase in chitosan content [38]. The electrostatic interaction could not maintain the stability of the emulsion when the concentration of chitosan exceeded 0.6%.

As Figure 5b shows, when the chitosan concentration was 0.6%, the particle size of the double-layer emulsion was 56.87 µm. With the addition of chitosan, the particle size of the double-layer emulsion increased, indicating that chitosan could be adsorbed on the surface of EWP-GP droplets to form a thicker interface layer [39]. When the chitosan concentration was greater than 0.8%, the particle size of the double-layer emulsion had no significant change (*p* > 0.05). This might be due to the saturation of chitosan adsorption on the surface of the emulsion when the concentration of chitosan exceeded 0.8%. When the concentration of chitosan was less than 0.6%, chitosan molecules were adsorbed between the droplets due to electrostatic interaction. A study also found that electrostatic interaction between egg-white protein and κ-Carrageenan led to an increase in bridging flocculation and particle size [40].

#### 2.2.2. Microstructures and Stability of Double-Layer Emulsion

**Figure 6:**
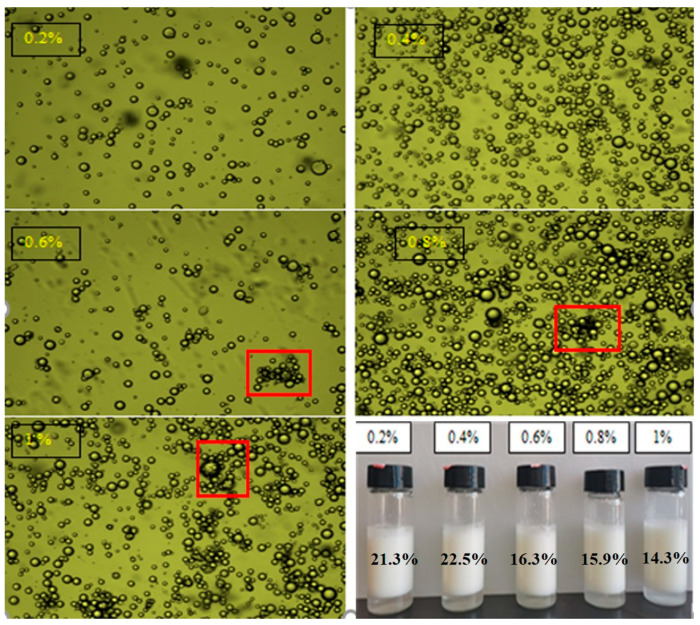
Microscopic image and macro view of double-layer emulsion. The graphs were collected at 100× magnification. (The red boxes point out that the emulsion drops gathered together. The creaming indexes of double-layer emulsions are labeled in the picture).

Figure 6 showed the microscopic image and macro view of double-layer emulsions with different chitosan concentrations. When the concentration of chitosan was 0.2% and 0.6%, the droplets dispersed uniformly. When the concentration of chitosan exceeded 0.6%, most of the drops gathered together. This might be due to the charge on the surface of the emulsion droplets being close to 0, and the electrostatic repulsion between the emulsion droplets decreased.

As was shown in Figure 6, the creaming index (CI%) of the double-layer emulsion decreased as the chitosan concentration increased from 0.4% to 1.0%, exhibiting better creaming stability. With the increase in chitosan concentration, chitosan molecules could form a film on the surface of the double-layer emulsion to prevent emulsion aggregation and improve the stability of the emulsion [41].

#### 2.2.3. Rheology Analysis

**Figure 7:**
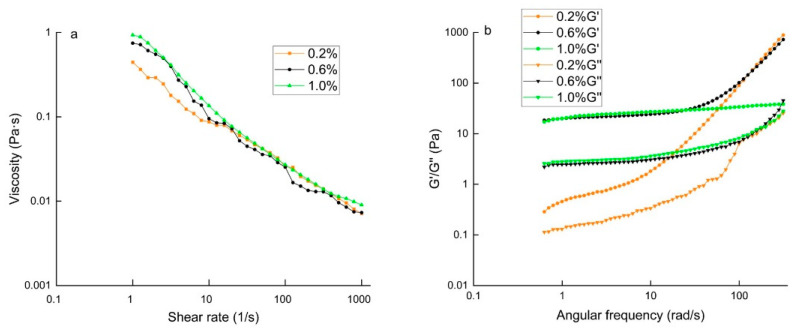
The viscosity of double-layer emulsions with various chitosan concentrations (**a**). Frequency dependence of the storage (G′) and loss (G″) moduli for double-layer emulsions with various chitosan concentrations (**b**).

The apparent shear viscosity of three emulsions decreased with an increase in shear rate (Figure 7a), indicating that they were non-Newtonian shear-thinning fluids [42]. At a low shear rate (less than 10/s), the apparent viscosity of the double-layer emulsion increased with the increase in chitosan concentration. This might be due to the internal resistance of the emulsion during flow, which hindered the free movement of the medium and led to a higher apparent viscosity [43]. With the increase in shear rate (more than 10/s), all of the droplets rearranged from disorder to order, and the apparent viscosity decreased. This might have occurred because any flocs or clumps in the emulsions had broken down at high shear rates. The shear viscosity hardly changed in the shear rate ranging from 10^2^ to 10^3^ s^−1^, indicating that the emulsions exhibited ideal fluid characteristics [44].

Storage modulus (G′) represents the elastic behavior of the emulsion network structure. The loss modulus (G″) represents the viscous behavior of the emulsion network structure. Figure 7b shows that G′ of each double-layer emulsion was much higher than that of G″ over the whole frequency range, suggesting that the gel network structure dominated by elasticity was formed in the emulsion [45,46]. When the chitosan concentration was 0.6%, the G′ of the double-layer emulsion was the highest. This phenomenon indicated that the interaction between droplets improved, and the ability to resist the deformation of the double-layer emulsion was strong. Meanwhile, the addition of chitosan molecules also improved the viscosity and contributed to the strength of the gel structure.

## 3. Materials and Methods

### 3.1. Materials and Reagents

The hen eggs were purchased from the Beijing CP Egg Industry Co., Ltd. (Beijing, China). Soybean oil (Yihai Kerry Arawana Holdings Co., Ltd.) was purchased from a local market (Wuhan, China). Glutamine transaminase (TGase, 100 U/g), chitosan, a Coomassie Brilliant Blue kit, Sodium Dodecyl Sulfate (SDS), Dithiothreitol (DTT), and ANS were purchased from Yuanye Bio-Technology Co., Ltd. (Shanghai, China).

### 3.2. The Preparation of Egg-White Protein Gel Particles

The egg-white protein gel particles (EWP-GPs) were prepared according to a previous method [47]. The egg-white was separated from fresh hen eggs and filtered with gauze to remove insoluble substances after stirring (1 h at 25 °C). The protein concentration of the solution was 10%, measured via Coomassie Brilliant Blue [48]. The EWP solution (30 mL) was ultrasonicated for 10 min at 120 W. The pH of the solution was adjusted to 7.0 with NaOH solution (1 mol/L) and HCl solution (1 mol/L). The EWP solution was stirred at 25 °C for 2 h and then reacted with TGase (20 U/g EWP) at 45 °C in a water bath for 2 h. The mixture of EWP and chitosan was heated at 90 °C for 40 min and then cooled in an ice bath immediately and maintained at 4 °C for 24 h. The gel particles were obtained, crushed, and diluted with deionized water. The gel particles were pre-homogenized for 2 min with a high-speed disperser (XHF-DY, Scientz, Ningbo, China) at 10,000 rpm. Finally, the EWP-GPs were obtained through homogenizing three times at 20,000 psi with a high-pressure micro-jet homogenizer (APV1000, APV Co., Crawley, UK).

### 3.3. Characterization of EWP-GPs

#### 3.3.1. Particle Size and Zeta Potential

In accordance with a previous method [26] and slightly modified, the particle size, PDI, and zeta potential of EWP-GPs and emulsions were determined via the particle electrophoresis instrument (Zetasizer Nano-ZS90, Malvern Instruments Ltd., Worcestershire, UK). The concentration of the EWP-GP solutions was diluted to 0.1% with deionized water to avoid multiple light scattering. The chitosan solution was diluted to 0.1% with deionized water and the zeta potential of the chitosan solution was determined through the particle electrophoresis instrument. Each sample was measured for three cycles and scanned 12 times per cycle.

#### 3.3.2. Wettability Measurement

In accordance with a previous method [49] with slight modifications, the wettability of EWP-GPs was evaluated through the three-phase contact angle (θ_o/w_) which was determined by using a VCA Optima system (AST Products Inc., Billerica, MA, USA). The fresh EWP-GPs were diluted to 0.1% and dropped onto the mica sheet. After drying at 25 °C for 16 h, the water droplets (2 µL) were added to the composite particles to determine the θ_o/w_. The θ_o/w_ was fitted to the Laplace-Young equation [50]. Each sample was repeated at least three times, and the average value was obtained.

#### 3.3.3. Emulsification

In accordance with a previous method [34] with slight modifications, the mixture of 30 mL EWP-GP solution (2%) and 10 mL soybean oil was treated via a high-speed homogenizer at 12,500 rpm for 2 min. The emulsion (50 µL) was removed from the bottom of the beaker, and then diluted 100 times with 0.1% (*w/v*) SDS. The absorbance of the emulsion at 500 nm was represented by the emulsification of the EWP-GPs.

#### 3.3.4. Intermolecular Force

The intermolecular force of EWP-GPs was determined by measuring the absorbance of the solution at the wavelength of 600 nm at 25 °C with a UV/VIS spectrophotometer (UV2000, UNICO (SHANGHAI) INSTRUMENT CO., LTD., China) via a previous means [51] with modifications. The pattern of intermolecular forces involved in forming and maintaining the EWP-GP structure was investigated by determining the absorbance of dispersion with different protein denaturants (6 mol/L urea, 0.5% (*w/w*) SDS, and 30 mmol/L DTT). The control group was not treated with any protein denaturant (urea, SDS, or DTT solutions). After 10 min of reaction, the EWP-GP solution was diluted to 0.1% (*w/w*) to remain in the linear region of absorbance. Each sample was measured at least three times in parallel, and the average value was taken. The transmittance (T) was calculated by the following formula:

(1) $$A=lg\frac{1}{T}$$

where *A* represented the absorbance of the solution and *T* represented the transmittance of the solution.

### 3.4. Egg-White Protein Chitosan Double-Layer Emulsion Preparation

In accordance with the previous study with slight modifications, the double-layer emulsion was prepared in two steps [7]. Firstly, the egg-white protein pickering emulsion (W_1_/O emulsion) was prepared with EWP-GP dispersion (4 wt.% EWP-GPs) and 40% (*v/v*) soybean oil, and the mixture was dispersed via a high-speed homogenizer at 15000 rpm for 2 min. Secondly, 0.2%, 0.4%, 0.6%, 0.8% and 1.0% (*v/v*) chitosan solutions (W_2_) were added to the Pickering emulsion, respectively. The mixture was dispersed via a high-speed homogenizer at 15,000 rpm for 2 min, and then egg-white protein-chitosan double-layer emulsion was obtained.

### 3.5. Double-Layer Emulsion Characterization

#### 3.5.1. Particle Size and Zeta Potential

The method to detect the particle size and zeta potential of the double-layer emulsion was the same as the method in Section 3.3.1. In addition, the emulsions were diluted to 0.1% with deionized water to avoid multiple light scattering. Each sample was measured for three cycles and scanned 12 times per cycle.

#### 3.5.2. Emulsion Microstructure

In accordance with the reported means [52] with modifications, the microstructure of the emulsion was observed through an optical microscope (SG–51, Shanghai optical instrument factory, Shanghai, China). The emulsion (20 µL) was placed on a microscope slide and covered by a cover slip. After balancing for 2 min, the microphotographs were collected. The graphs were collected at 100× magnification.

#### 3.5.3. Creaming Index (*CI*)

The creaming index was measured via a reported method [23] with modifications. Fresh emulsion (8.0 mL) was added to the test vials and sealed with lids immediately after preparation. The height of the serum phase at the bottom (*Hs*) and the total height of emulsions (*Ht*) were recorded at fixed intervals. The experiment was carried out at 25 °C and the percentage of *CI* (%) was calculated using the following equation:

(2) $$CI\left(\%\right)=\frac{Hs}{Ht}\times 100$$

where *Hs* represented the height of the serum phase and *Ht* represented the total height of emulsions.

#### 3.5.4. Rheology Measurement

The rheology of the double-layer emulsions was performed with a Discovery DHR-2 Rheometer (TA Instruments, New Castle, DE, USA) by a previous means [46] with modifications. The samples were placed between two parallel plates with a diameter of 40 mm at 25 °C. The static shear rheological properties of the emulsions were measured at a shear rate from 0.1 to 100 rad/s. The storage modulus (G′) and loss modulus (G″) of the emulsions were recorded as a function of frequency from 0.1 to 16 Hz.

### 3.6. Statistical Analysis

Data were presented as mean ± standard deviation (SD) of three independent experiments. The statistical analysis was assessed using one-way analysis of ANOVA with Duncan’s multiple range test. *p*-values < 0.05 were considered as statistically significant.

## 4. Conclusions

In this study, the EWP-GPs and EWP–chitosan double-layer emulsion were successfully prepared. The results showed that ultrasonic treatment combined with TGase treatment could significantly improve the surface hydrophobicity and stability of EWP-GPs. The optimal EWP-GPs had a smaller particle size, higher dispersity, better emulsification, and good wettability. The intermolecular force results showed that the hydrogen bond was the main force to maintain the stability of EWP-GPs. Based on these results, the EWP-chitosan double-layer emulsion was prepared and characterized. The double-layer emulsion had a small particle size and the droplets were dispersed uniformly, suggesting high stability. The rheological properties of the double-layer emulsion indicated that the gel network structure dominated by elasticity was formed in the emulsion. The prepared EWP-chitosan double-layer emulsion performed with high stability and good creaming stability. In a further study, we could study the delivery functions of the EWP-chitosan emulsion. It could provide a novel food-grade delivery system for bioactive substances, such as β-carotene and polyphenol.

## Data Availability

Not applicable.

## Abbreviations

EWP-GPs	Egg-white protein gel particles
TGase	Transglutaminase
PDI	Polymer dispersity index
CI	Creaming index
